# A3D database: structure-based predictions of protein aggregation for the human proteome

**DOI:** 10.1093/bioinformatics/btac215

**Published:** 2022-04-21

**Authors:** Aleksandra E Badaczewska-Dawid, Javier Garcia-Pardo, Aleksander Kuriata, Jordi Pujols, Salvador Ventura, Sebastian Kmiecik

**Affiliations:** 1 Department of Chemistry, Iowa State University, Ames, 50011 IA, USA; 2 Institut de Biotecnologia i de Biomedicina (IBB) and Departament de Bioquímica i Biologia Molecular, Universitat Autònoma de Barcelona, 08193 Barcelona, Spain; 3 Biological and Chemical Research Center, Faculty of Chemistry, University of Warsaw, Warsaw 02-093, Poland

## Abstract

**Summary:**

Protein aggregation is associated with many human disorders and constitutes a major bottleneck for producing therapeutic proteins. Our knowledge of the human protein structures repertoire has dramatically increased with the recent development of the AlphaFold (AF) deep-learning method. This structural information can be used to understand better protein aggregation properties and the rational design of protein solubility. This article uses the Aggrescan3D (A3D) tool to compute the structure-based aggregation predictions for the human proteome and make the predictions available in a database form. In the A3D database, we analyze the AF-predicted human protein structures (for over 20.5 thousand unique Uniprot IDs) in terms of their aggregation properties using the A3D tool. Each entry of the A3D database provides a detailed analysis of the structure-based aggregation propensity computed with A3D. The A3D database implements simple but useful graphical tools for visualizing and interpreting protein structure datasets. It also enables testing the influence of user-selected mutations on protein solubility and stability, all integrated into a user-friendly interface.

**Availability and implementation:**

A3D database is freely available at: http://biocomp.chem.uw.edu.pl/A3D2/hproteome. The data underlying this article are available in the article and in its online supplementary material.

**Supplementary information:**

Supplementary data are available at *Bioinformatics* online.

## 1 Introduction

In July 2021, a database of highly accurate structure predictions for the human proteome was published (Tunyasuvunakool *et al.*, 2021). The predictions computed using the newly developed neural network model AlphaFold (AF), were shown to be competitive with experimental structures (Jumper *et al.*, 2021).

Here, we have constructed the AGGRESCAN3D (A3D) Database by computing the aggregation propensity of the human protein models from the AF database. The A3D is a structure-based predictor of surface-exposed aggregation-prone regions. The A3D algorithm exploits the information of 3D atomic models to compute the structurally corrected aggregation values (A3D score) for each amino acid (Kuriata *et al.*, 2019a, b; Pujols *et al.*, 2018; Zambrano *et al.*, 2015). A3D can predict the effect of mutations on protein stability and aggregation propensity, as well as suggest solubility-enhancing mutations. This algorithm has been employed to study the constraints imposed by aggregation on protein evolution (Carija *et al.*, 2019), to diagnose the functional impact of genetic mutations (Seaby and Ennis, 2020), to predict the aggregation of the SARS-CoV-2 proteome (Flores-León *et al.*, 2021), to assist the design of novel nanomaterials (Gil-Garcia and Ventura, 2021) or to engineer the solubility of therapeutic proteins (de Aguiar *et al.*, 2021; Gil-Garcia *et al.*, 2018) among many other applications.

## 2 A3D database features

The A3D database integrates A3D analysis for 23391 predicted structures of the human proteome from the AF database. The content of the A3D database can be queried by UniProt ID, Gene or protein name (see Movie S1 in Supplementary Information for the short tutorial). Clicking the selected protein target in the results list leads to the subpage of an entry in the A3D DB. The A3D predictions are presented in a series of tabs that link to pages containing: (i) the project details, (ii) an interactive A3D score profile and annotation of transmembrane regions (if applicable), (iii) a detailed table containing A3D scores and AF structure prediction confidence scores (pLDDTs), (iv) the structural information, (v) customizable calculations and (vi) an image gallery.

**Fig. 1.**
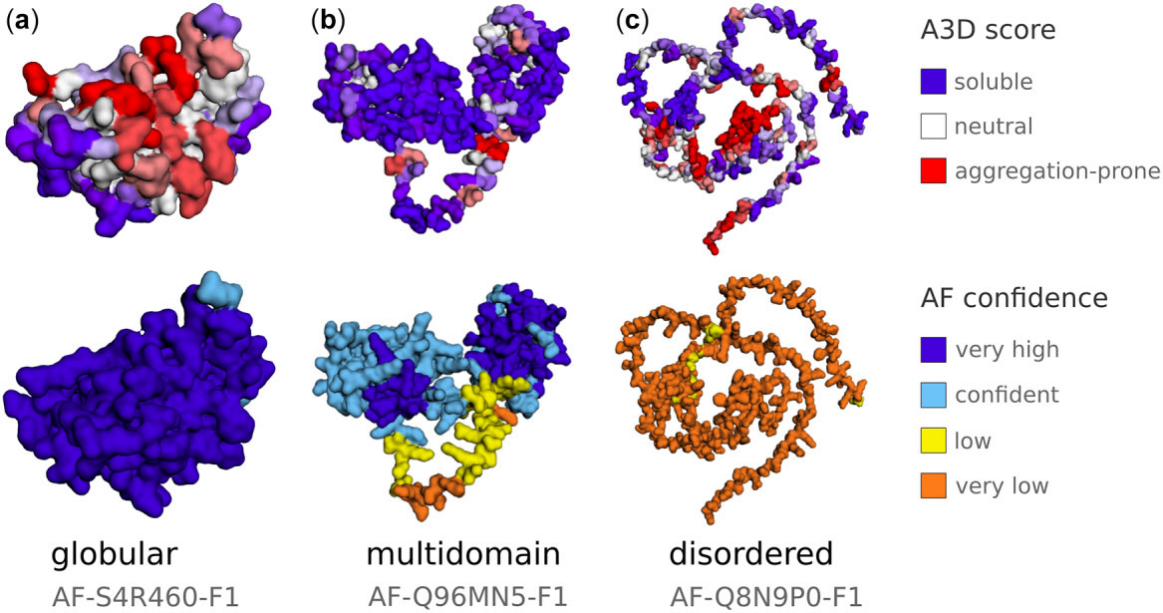
Examples of protein model visualizations from the A3D database. For each database entry, under the Structure tab, two protein copies are presented colored according to (i) the A3D score and (ii) the AlphaFold (AF) model confidence score. The A3D score is visualized in shades from dark blue (highly soluble residues, score < −2.5), through white (no predicted influence on aggregation properties), to dark red (aggregation-prone residues, score > +2.5). The AF per-residue confidence score (pLDDT) is presented in dark blue (very high confidence, pLDDT > 90), light blue (confident, 90 > pLDDT > 70), yellow (low confidence, 70 > pLDDT > 50) and orange (very low confidence, pLDDT < 50). Note that pLDDT < 50 is a reasonably strong predictor of disorder (Tunyasuvunakool *et al.*, 2021), which suggests that a particular region may be unstructured as a linker between domains (see **b**) or as an inherently disordered domain (see **c**). (**a**) An example of a globular protein predicted with high confidence is shown (A color version of this figure appears in the online version of this article)

In the Structure tab, protein structures can easily be visualized and analyzed interactively. Two different models are presented for each entry (see Fig. 1). The top model reports on the A3D score (A3D score, a per-residue estimate of aggregation propensity, see Fig. 1), while the bottom model depicts the AF pLDDT score (pLDDTs score, a per-residue estimate of structure prediction confidence, see Fig. 1) (Tunyasuvunakool *et al.*, 2021). Note that low pLDDTs might result in misleading A3D predictions because often they correspond to protein regions that are either more exposed or sheltered in the model than in their native/natural conformation (see Supplementary Information). Because of that, we performed A3D analysis using three different AF models for each protein entry: the full-length protein model and two additional models in which residues with pLDDT < 70 or residues with pLDDT < 50 were removed (see Supplementary Information). Access to these two additional models is provided in the Custom Jobs tab. This subpage allows also to submit a new job to the A3D server with individual residues removed via residue editor or according to a user-selected pLDDT cutoff (see Notes in Supplementary Information). In addition, a mutation editor allows the introduction of one or multiple mutations in another custom A3D job, where the predicted changes in solubility and stability can be retrieved.

In summary, the A3D database can be helpful in the study and redesign of human proteins' solubility (also in combination with other human proteome predictions; Prabakaran *et al.*, 2021). It also allows investigating correlations between structural aggregation propensity and protein function, stability, architecture, location, abundance, lifetime or essentiality at the proteome level. In Supplementary Information, we illustrate and discuss the utility of the database with selected case reports.

## Funding

A.E.B-D. received financial support from Roy J. Carver Charitable Trust and Iowa State University Foundation. This work was supported by the Spanish Ministry of Science and Innovation (MICINN) [PID2019-105017RB-I00 to S.V.], by ICREA, ICREA-Academia 2019, and by EU [PhasAge/H2020-WIDESPREAD-2020-5 to S.V.]; the Spanish Ministry of Science and Innovation with a Juan de la Cierva Incorporacion [IJC2019-041039-I to J.G.-P.]; S.K. acknowledges funding from the National Science Centre, Poland [2020/39/B/NZ2/01301].

*Conflict of Interest*: none declared.

## Supplementary Material

btac215_Supplementary_Data
