# Supplementary material for: A3D database: structure-based predictions of protein aggregation for the human proteome
Source: Bioinformatics. 2022 Apr 21;38(11):3121–3. doi: 10.1093/bioinformatics/btac215 (PMC9746890; doi:10.1093/bioinformatics/btac215)
Supplement: btac215_Supplementary_Data [file btac215_supplementary_data.docx]

**SUPPLEMENTARY INFORMATION to the manuscript:**

**“A3D Database: Structure-based Protein Aggregation Predictions for the Human Proteome”**

**Movie S1.** A short tutorial on using the Aggrescan3D (A3D) database.

1. **Methods**
   1. **A3D analysis**

We performed an A3D analysis for 23391 structures of the Human proteome available for download from the AF database at <https://ftp.ebi.ac.uk/pub/databases/alphafold/latest/UP000005640_9606_HUMAN_v2.tar>. For each case, we tested the aggregation properties of three variants. The first was the original AF-predicted structure, while the second and third were modified structures deficient in residues with a pLDDT score lower than 70 and 50, respectively. We run all jobs through the RESTful service of Aggrescan3D 2.0 web server with default A3D settings, i.e., with 10 Å distance of aggregation prediction and FoldX-based energy minimization for stability calculations.

All results are freely available online in the A3D database http://biocomp.chem.uw.edu.pl/A3D2/hproteome

- 1. **Database construction**

The AF Database collects 23391 protein structure predictions assigned to the *Homo sapiens* proteome, corresponding to 20504 unique UniProt entries [(Jumper *et al.*, 2021)](https://paperpile.com/c/2SWuDF/qN6VB). This set covers well the subset of human proteins with reviewed status, which are longer than 16 amino acids. The extremely long sequences (longer than 2700 residues and up to 34350) were split into overlapping fragments and modeled independently by AF Database researchers. As a result, the AF downloadable repository provides multiple structure predictions, a few to several dozen, for a particular UniProt identifier. Such UniProt entries are not available at all in the interactive interface of the AF Database - they are only available for download. On the day we downloaded the dataset (4 August 2021), a single structural model was assigned for each AF entry.

The entries in the A3D database intentionally correspond to the individual files/structure fragments available in the AlphaFold database. At the same time, we provide the user with a high level of control over the fragments corresponding to a single UniProt sequence. When a single UniProt sequence is split into multiple entries in the A3D database, all of them are listed when the database is queried for a given UniProt ID, e.g., searching for O95613 corresponds to 11 AlphaFold predictions. Moreover, we indicate precisely the region of the sequence (4th column) to which a given fragment of the structure corresponds (see **Figure S1**).


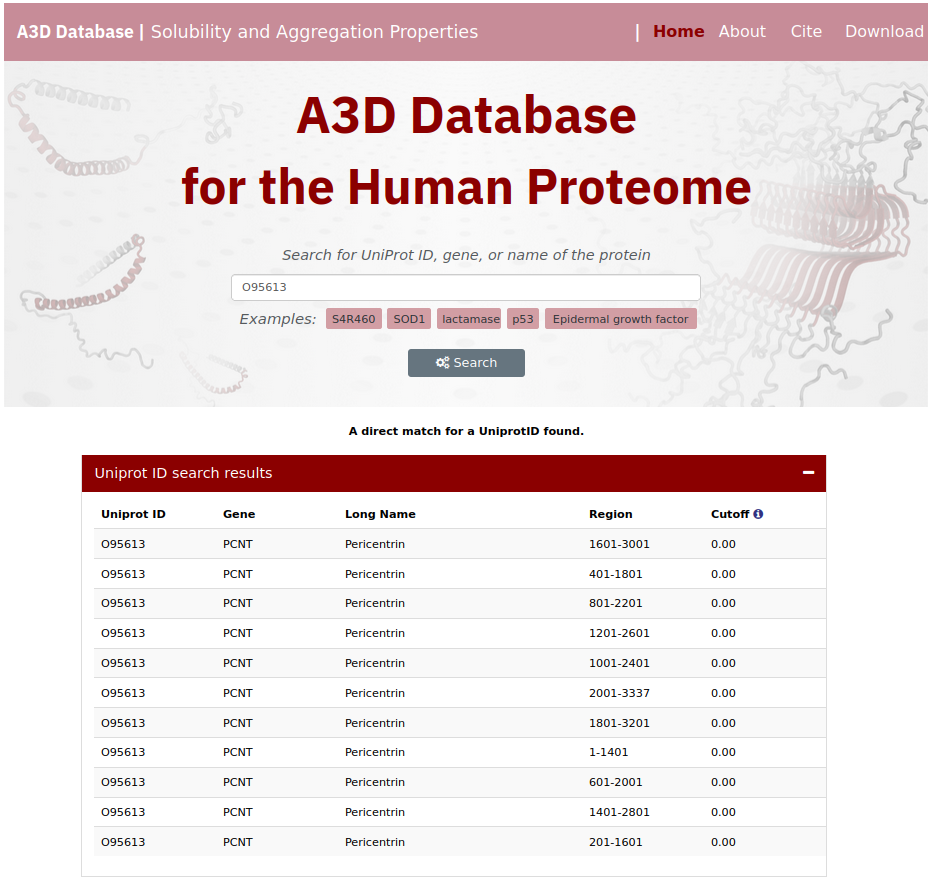


**Figure S1.** A3D database home page with search results by *O95613* UniProt ID. The fourth column defines the region of the sequence that corresponds to the predicted structure from a given entry in the AF Database. A3D entries correspond to individual structures in the AF Database even if they only cover a fragment of the UniProt sequence.

***Features of A3D Database***

Selecting a hit for a particular region takes the user to the page of that entry in the A3D database where the results of Aggrescan3D analysis are collected. Among 6 available tabs, *Project details* is opened by default for the given entry (see **Figure S2**). There at the top the user will find the assigned ‘*Project name*’, e.g., AF-O95613-F7, that corresponds (in this case to the seventh, -F7) AlphaFold prediction of the fragment of the UniProt sequence.


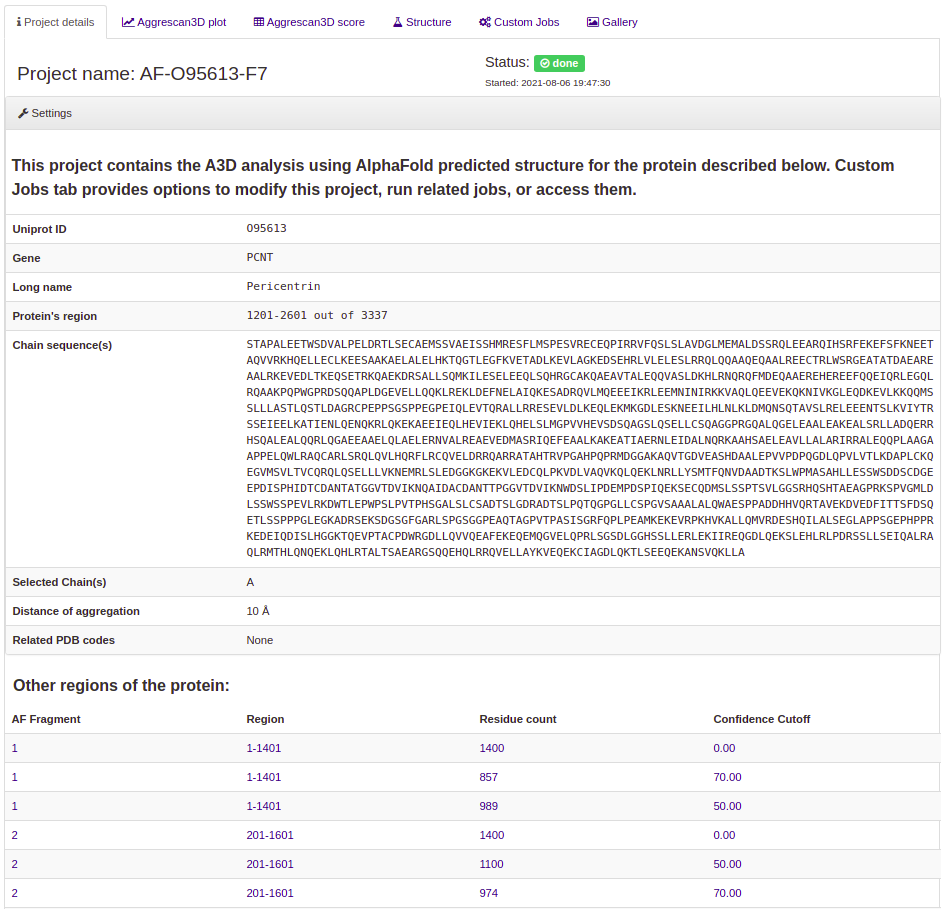


**Figure S2.** A3D database results page of *Projects details* tab for *AF-O95613-F3* entry.

For all the proteins, we collected the additional information that can be used in database searching. They include UniProt identifier (*e.g., P01133*), AlphaFold identifier for structural entry (e.g., *AF-P01133-F1*), gene (e.g., *EGF*), common short name (e.g., *Urogastrone*), and long descriptive name (e.g., *Pro-epidermal growth factor*). The contents of the first three fields in the results page (*‘Uniprot ID’. ‘Gene’, ‘Long name’*) correspond exactly to the query keys that can be used to search for a given entry in the A3D Database.

The ‘*Protein's region*’ field provides the range of amino acids and the total length of the original UniProt sequence (e.g., 1201-2601 out of 3337 amino acids). For user convenience, we added information about the full length of the original sequence. In addition, for sequences split into fragments within the AF Database, we provided the region corresponding to the predicted structure. This information is not even available in the AlphaFold database. We determined this as part of our analysis to make it much easier to identify a domain of interest to the user. In total, the A3D database contains over 23 thousand structural deposits, corresponding to 20504 unique UniProt IDs (individual proteins, because for some cases, the original sequence was split into fragments).

The next field ‘*Chain sequence(s)*’ contains the exact amino acid sequence of the fragment in one-letter notation for the user convenience. Furthermore, for multi-fragment entries corresponding to a single UniProt ID, an additional '*Other regions of the protein*' section appears in which all other related entries are listed and linked. The user may choose to open them simultaneously in separate tabs or new browser windows on a larger screen (to view multiple structures all at once for comparison). This is the best solution for multi-entry Uniprot IDs that could be worked out while preserving the structure and efficiency of the A3D Server and Database.

Further, for a given A3D entry we have added an additional field *'Related PDB codes'* which contains a list of PDB codes assigned to the given UniProt ID. The PDB codes were taken directly from the UniProt database. Performing Aggrescan3D analysis for all reference PDB codes is beyond the scope of this work. However, if necessary, the user can easily submit the request of such analysis directly on the A3D server using the PDB codes from the pre-defined list for each entry.

When a single UniProt ID corresponds to multiple entries with a prediction structure of sequence fragments, the '*Other regions of the protein*' section appears. Because the predicted structures vary considerably in confidence level (pLDDT), we also report for each entry the number of residues that remain in the structure after removing residues below the two predefined cutoffs, a threshold of 70 and 50. That can help to screen out underpredictions for user-defined A3D analysis. The results of the A3D analysis for such customized criteria are pre-calculated and available from the *Custom Jobs* tab or directly by clicking on a hit from the list.

***Annotation of the membrane proteins***

The membrane proteins have specific physicochemical properties on their surface, which can significantly bias the A3D predictions due to its sensitivity to highly hydrophobic fragments. Thus, for the total of 5156 transmembrane and intramembrane proteins, detected in the Human proteome via UniProt annotation, we performed a more detailed identification of membrane regions. For this task, we used the TOPCONS server [(Tsirigos *et al.*, 2015)](https://paperpile.com/c/2SWuDF/xH7H), which given the amino acid sequence specifies the membrane segments as consensus from five different topology prediction algorithms: OCTOPUS, Philius, PolyPhobius, SCAMPI, and SPOCTOPUS. As a result, we provide users with high-quality graphs that facilitate visual interpretation and possible rejection of transmembrane fragments from A3D analysis results. For user convenience, the annotation of transmembrane regions (for cases where this is applicable) has been placed in the separate *Transmembrane regions* tab (see **Figure S3**).

**
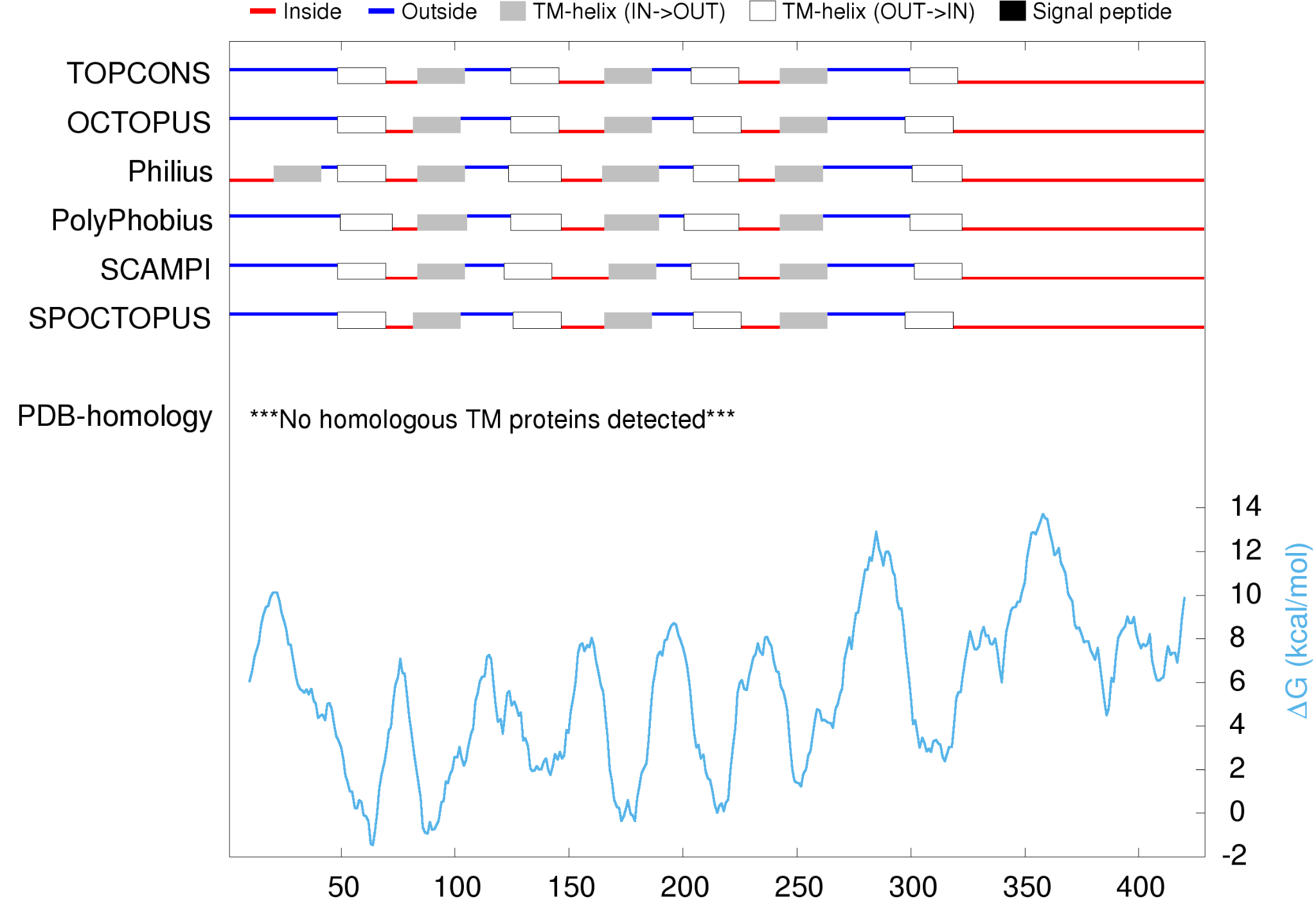
**

**
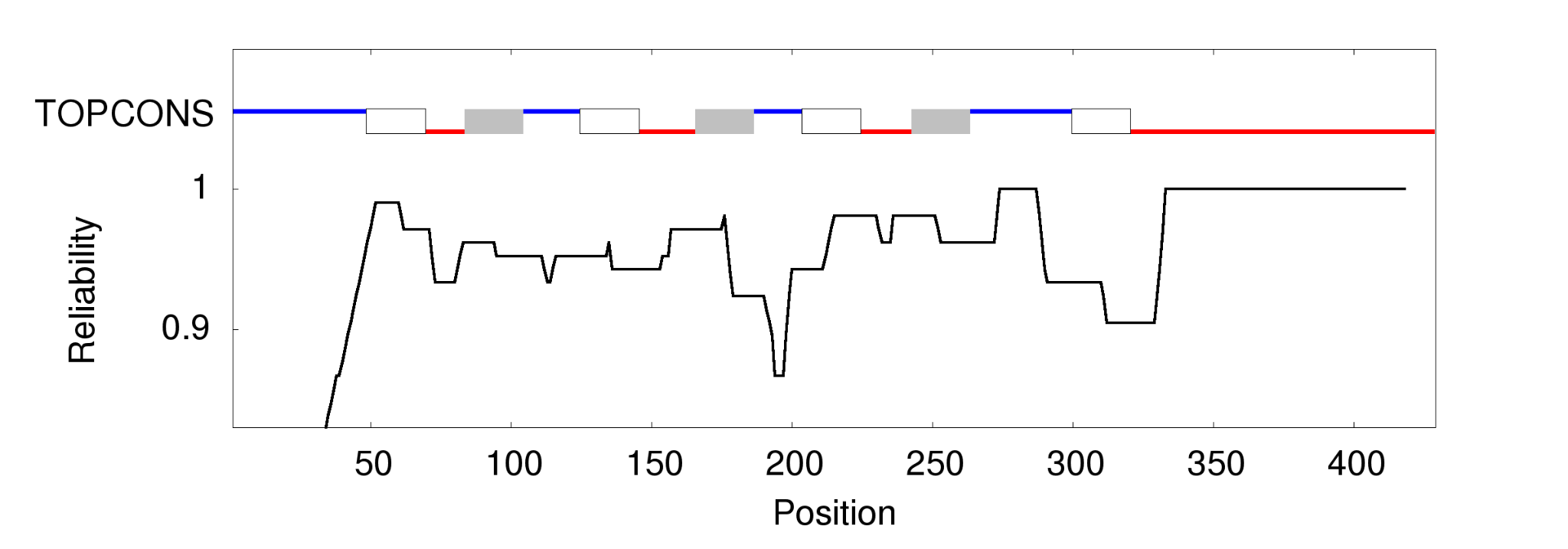
**

**Figure S3.** A3D database results page of *Transmembrane regions* tab for Q8N3F9 entry (integral membrane protein GPR137C; transmembrane 7 superfamily member 1-like 2 protein). The upper panel shows predictions from different algorithms, the lower panel presents prediction reliability (for details [(Tsirigos *et al.*, 2015)](https://paperpile.com/c/2SWuDF/xH7H)).

***Download options***

The unified and integrated metadata accompanied by referencing identifiers in the A3D database is available for download in CSV format from the Download tab of the A3D database webpage (see **Figure S4**).


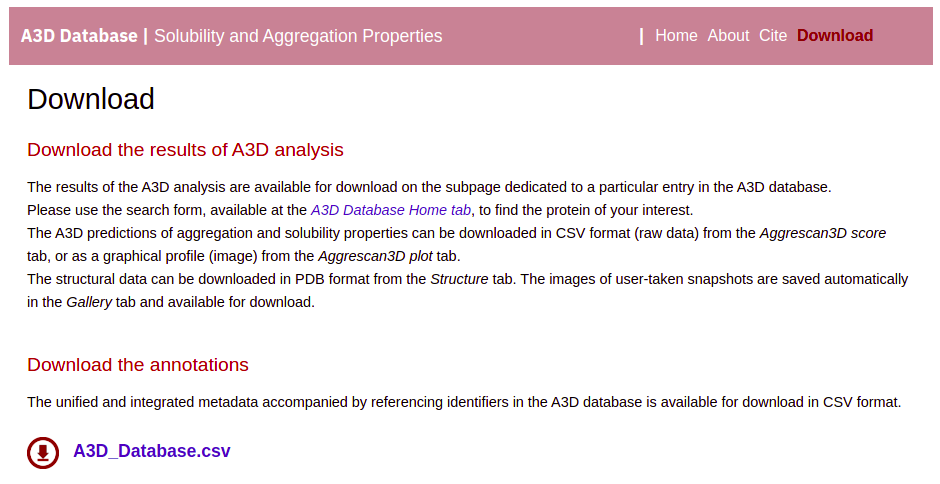


**Figure S4.** The Download subpage in the main database view enables downloading all annotations and A3D scores for all entries at once. The About subpage, in section *‘Downloading results through a REST API’* provides instructions and sample python script for downloading the results for a particular entry or custom job submitted to A3D server.

Further, the results of A3D analysis for each pre-calculated entry in the A3D Database, as well as for any user-defined custom job can be downloaded via the REST API. Any job can be identified by an unique identifier called a *job_id*, which can be found in the address of the web browser when viewing the job (e.g., http://biocomp.chem.uw.edu.pl/A3D2/hproteome_job/65417ebb9b66183/ would mean that the job ID = 65417ebb9b66183). The list of *job_id* for all predefined entries in the A3D Database is in the file *A3D_Database.csv* available for download as discussed in the section above. A sample Python script for an automatic download is provided in the About subpage in the main view of A3D Database.

**2. Example cases**

Below we investigate two example cases. In the first example, we analyzed the structure of the human Copper-Zinc Superoxide Dismutase (SOD1), for which a variety of mutations underlie the formation of protein deposits in familial amyotrophic lateral sclerosis [(Deng *et al.*, 1993)](https://paperpile.com/c/2SWuDF/qVYwa). Most mutations are neutral in terms of sequential intrinsic aggregation propensity but impact the stability of the native dimer structure, favoring its dissociation into the monomeric subunits. A comparative analysis of the structural aggregation propensity of the monomer was done with A3D using either the experimental PDB coordinates or its equivalent AF-derived model (Figure 2). In both cases, A3D detected the presence of a strong S-APR that overlaps with the dimerization interface (**Figure S5**). As a consequence this aggregation-prone region is protected within the homodimer but exposed to solvent in the monomer, explaining why mutations favoring dissociation promote SOD1 deposits [(Elam *et al.*, 2003)](https://paperpile.com/c/2SWuDF/xo0jY).


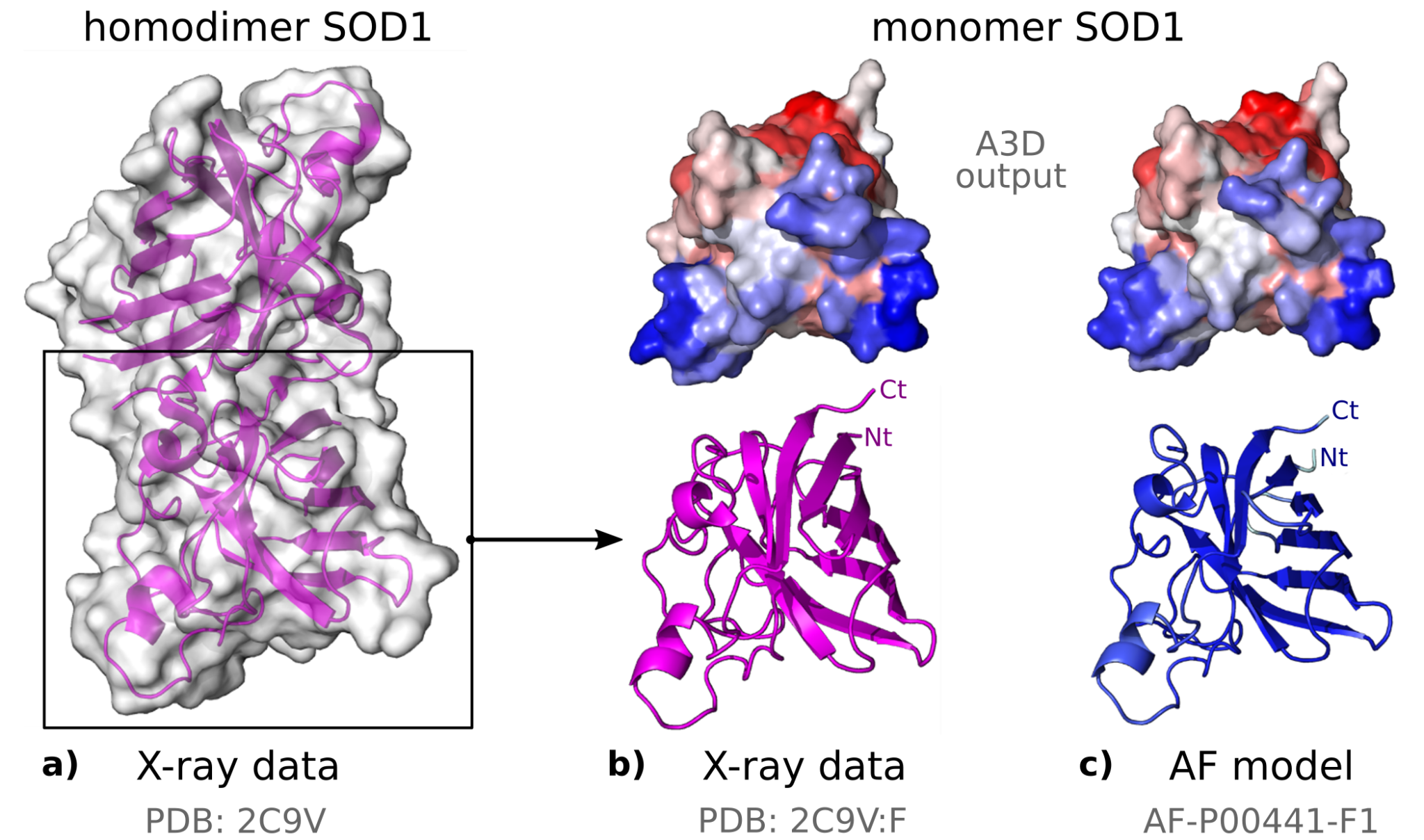


**Figure S5**. Comparison of the aggregation propensity of human Copper-Zinc Superoxide Dismutase (SOD1) calculated with A3D using PDB data and the equivalent AlphaFold2 model. a) Crystal structure of SOD1 homodimer (PDB ID: 2C9V (Strange et al. 2006)) b) SOD1 X-ray structure (PDB ID: 2C9V:F, bottom panel) and its A3D analysis (top panel). C) SOD1 AlphaFold model (AF-P00441-F1, bottom panel) and its A3D analysis (top panel). A3D analysis is presented using a coloring scheme in a gradient from blue (high solubility) to white (no impact on protein aggregation) to red (high aggregation propensity).

In a second example, we analyzed Beta-2 microglobulin (β-2 m), a protein whose aggregation is associated with dialysis-related amyloidosis [(Gejyo *et al.*, 1985)](https://paperpile.com/c/2SWuDF/AGIF). Residue W60 is heavily involved in both functional contacts in the I major histocompatibility complex contacts and aberrant intermolecular interactions leading to the protein aggregation upon β2m dissociation. Accordingly, the non-natural W60G mutation protects β2m from aggregation by reducing the exposed aggregation-prone surface. Incorporating the N83V mutation on top of the W60G change reverts its protective effect by turning a soluble region in the protein surface into an aggregation-prone one. In contrast, the V85E mutation alone had an opposite effect, precluding aggregation by turning a hydrophobic surface patch into a polar one. Modeling of β-2 m WT and the three mutants with AF and its subsequent analysis with A3D allowed us to recapitulate all this experimental structural evidence [(Camilloni *et al.*, 2016)](https://paperpile.com/c/2SWuDF/WguV) (Figure S6). The average A3D scores for WT, V85E, W60G and W60G/N83V AF-models are -1.26, -1.37, -1.30 and -1.25, respectively, in good agreement with their relative experimental aggregation propensities.


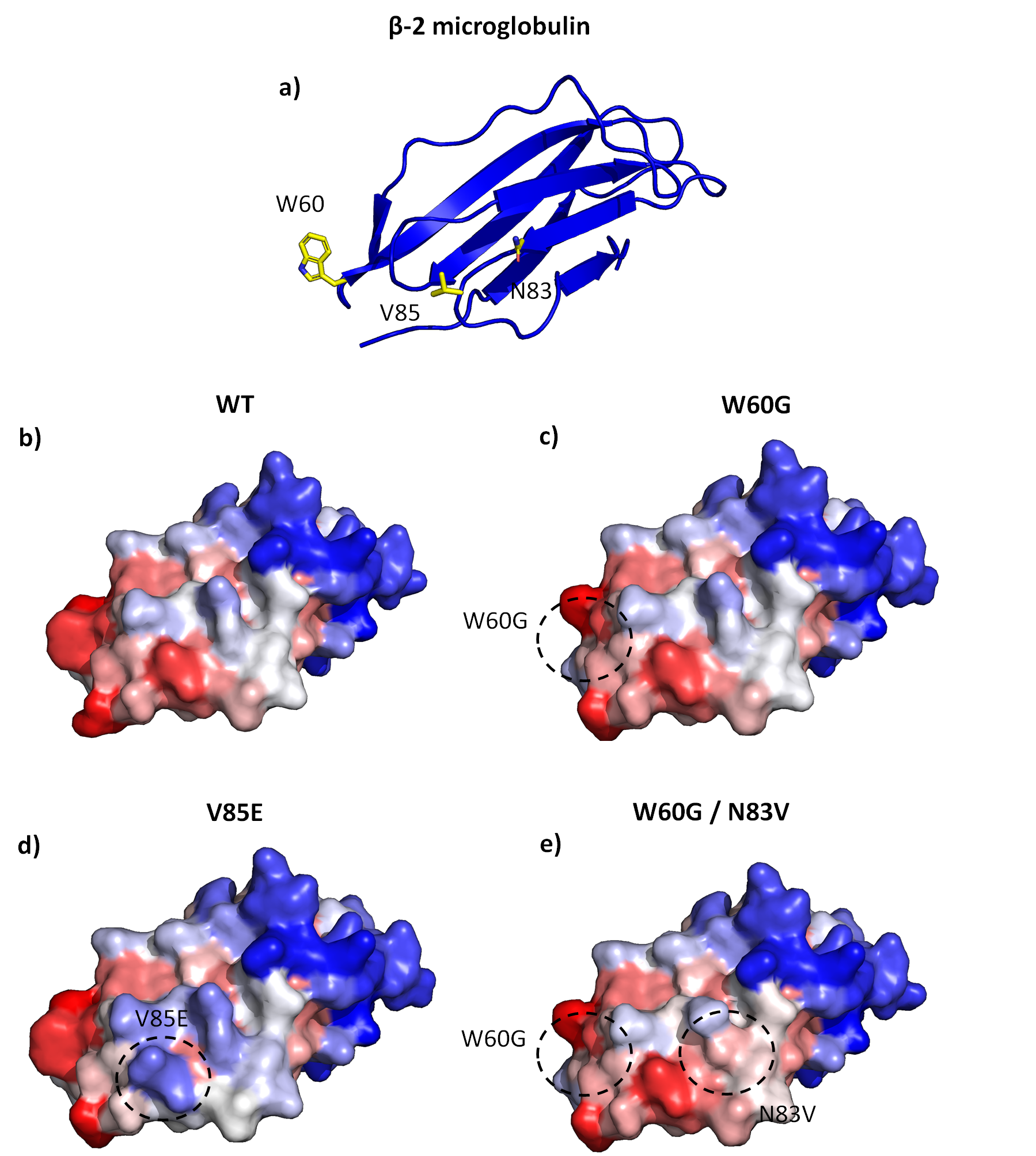


**Figure S6**. Aggregation propensity of human beta-2 microglobulin (β-2 m) predicted with A3D. a) AlphaFold structural model of β-2 m generated with AF (AF-P61769-F1, residues 21-118) in a ribbon representation. The side chains of the mutated residues are shown as yellow sticks. b) Aggregation propensity of wild-type β-2 m (WT) calculated with A3D. c-e) Comparison of the aggregation propensity of three different β-2 m mutational variants. Protein surfaces are colored according to the A3D score.

- 1. **Prediction of the impact of mutations on protein solubility and stability**

A3D has been shown to be accurate in predicting the changes in the solubility of globular proteins upon mutation [(Zambrano *et al.*, 2015)](https://paperpile.com/c/2SWuDF/tGjOh). We compared the performance of A3D when modeling the impact of mutations on the solubility on top of either the experimental structures and the equivalent AF-derived models, using the mutation editor in the A3D database. To this aim, we selected a reduced set of structurally and sequentially unrelated proteins (**Table S1** and **Figure S7**). The predictions turned out to be accurate and coincident for all the proteins, independently of the kind of structural input we used. In addition to changes in solubility, A3D provides the impact of the selected mutations on protein stability, as calculated by FoldX [(Schymkowitz *et al.*, 2005)](https://paperpile.com/c/2SWuDF/1UBEi). Importantly, it has been shown that the quality of AF models is sufficient to predict protein solubility changes upon mutation using FoldX[(Akdel *et al.*, 2021)](https://paperpile.com/c/2SWuDF/2nMSW).


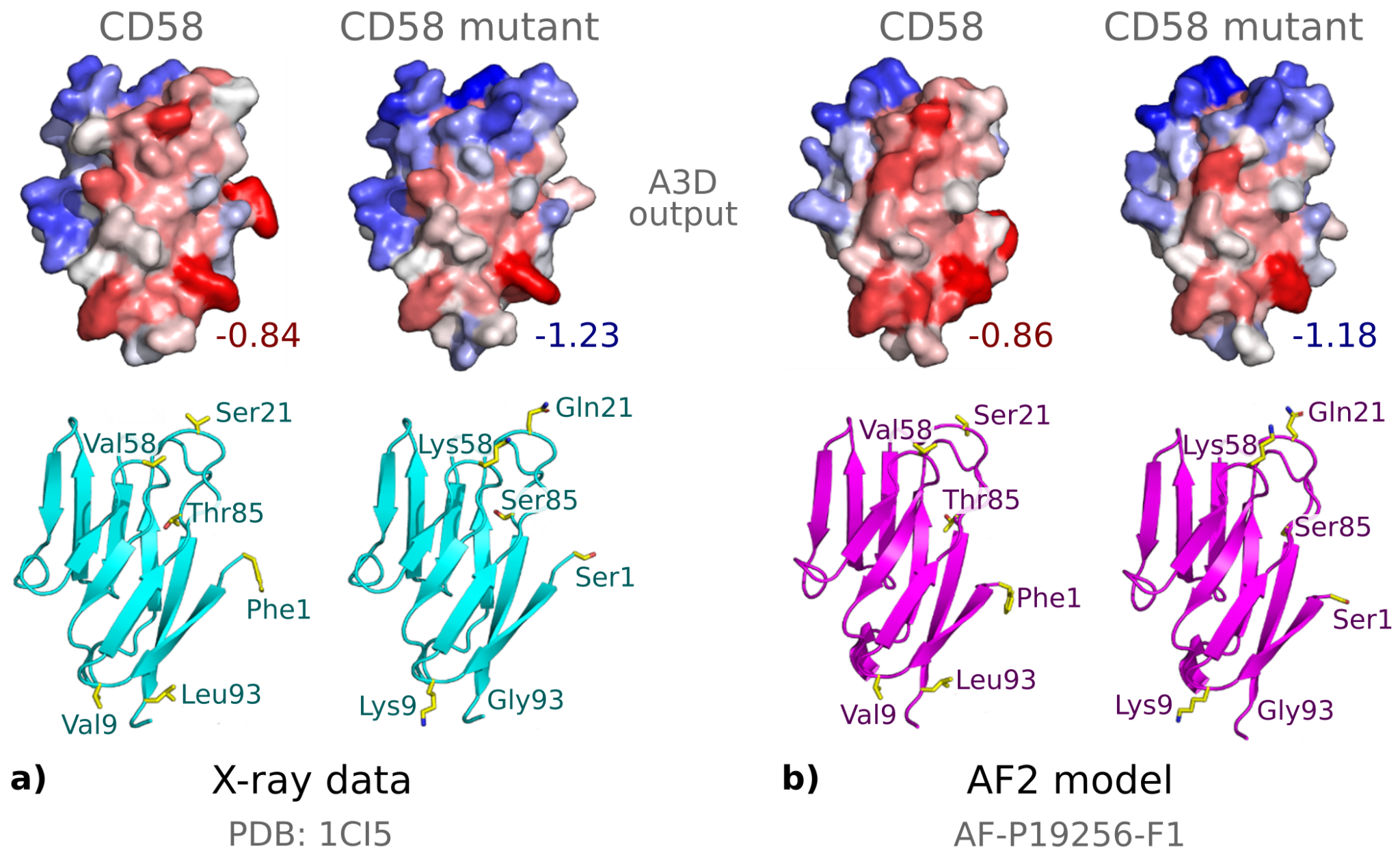


**Figure S7**. Comparison of the performance of A3D in predicting the changes in the solubility of Lymphocyte function-associated antigen 3 (CD58) between a) the experimentally determined structure (PDB ID: 1CI5 (Sun *et al*.,1999), colored in cyan) and b) the Alphafold2-derived model (AF-P19256-F1, colored in magenta). The top row in both panels shows A3D analysis for the wild-type CD58 (WT) and its solubility-optimized mutant (see Table S2 for details), respectively. For both sources of structural data, the introduced mutations (F1S, V9K, S21Q, V58K, T85S, L93G) result in a more soluble CD58 variant. The calculated average A3D scores improve from -0.84 to -1.23 for X-ray structure, and from -0.86 to -1.18 for the AF2 model. In all the cases, surface representations are colored according to the predicted A3D score, as defined in **Figure 1**.

| **Table S1.** Randomly selected case study AF structures, grouped alphabetically by their Uniprot identifier. |
| --- |
| A0A0B4J263, A0A1B0GVH4, A0A7I2V3D3, A6NC57, A6NKH3, A8MZ26, J3KSC0, O14602, O15091, O43278, O60258, O75044, O75626, O94901, O95613, P00748, P02776, P06400, P09382, P0CJ87, P10243, P13611, P16989, P20810, P23458, P28360, P34896, P40617, P46779, P49792, P52566, P55884, P60842, P63173, P84550, Q02224, Q05639, Q0VDD8, Q13151, Q13753, Q14533, Q15149, Q15831, Q17RW2, Q3LHN1, Q4UJ75, Q5EE01, Q5SVQ8, Q5TBA9, Q5VUJ9, Q68D91, Q6MZM9, Q6PF06, Q6UXY1, Q6ZRI0, Q709C8, Q7Z2R9, Q7Z6R9, Q86UW9, Q86YS6, Q8IWC1, Q8IYW2, Q8N2C9, Q8N660, Q8N9P0, Q8NDA2, Q8NFW1, Q8TC99, Q8TEV8, Q8WWM1, Q8WZ42, Q92626, Q969T7, Q96DA0, Q96GQ7, Q96K80, Q96MN5, Q96Q35, Q99218, Q99973, Q9BSV6, Q9BWX1, Q9BZ19, Q9GZY1, Q9H2X6, Q9H772, Q9HB66, Q9NPE2, Q9NRE2, Q9NV70, Q9NYC9, Q9P1Y6, Q9UBH0, Q9UHL9, Q9UKL6, Q9UND3, Q9Y243, Q9Y3F1, Q9Y5P4 and S4R460 |

**Table S2**. Comparison of the performance of AGGRESCAN3D to predict changes in protein solubility using experimentally determined structures and their equivalent AlphaFold2- derived models. For each protein presented in Table S1, the changes in solubility upon mutation and expression have been previously characterized elsewhere [(Adachi *et al.*, 1993; Sun *et al.*, 1999; Ito and Wagner, 2004; Sim and Sim, 1999; Nasreen *et al.*, 2006; Murphy and Tsai, 2007)](https://paperpile.com/c/2SWuDF/jybo+kCcT+8SAh+K51c+r7wD+2zbF).

|  |  |  | **AGGRESCAN 3D PREDICTION** | | | |  |
| --- | --- | --- | --- | --- | --- | --- | --- |
| **Protein Name** | **Mutation/s** | **Solubility** | **PDB code** | **Result** | **AF code** | **Result** | **Ref** |
| Hemoglobin subunit beta | Glu6Leu | Decreased | 2D60 (B) | TN | AF-P68871-F1 | TN | [1] |
|  | Glu6Phe | Decreased | 2D60 (B) | TN | AF-P68871-F1 | TN | [1] |
|  | Glu6Trp | Decreased | 2D60 (B) | TN | AF-P68871-F1 | TN | [1] |
| CD58 | Phe1Ser  Val9Lys  Val21Gln  Val58Lys  Thr85Ser  Leu93Gly | Increased | 1CI5 (A) | TP | AF-P19256-F1 | TP | [2] |
| Translation initiation factor eIF2a | Ala27Gln  Leu46His  Val71Lys | Increased | 1Q8K (A) | TP | AF-P05198-F1 | TP | [3] |
| Interleukin 1 Beta | Leu10Asn | Decreased | 9ILB (A) | TN | AF-P01584-F1 | TN | [4] |
|  | Leu10Asp | Decreased | 9ILB (A) | TN | AF-P01584-F1 | TN | [4] |
|  | Lys97Gly | Decreased | 9ILB (A) | TN | AF-P01584-F1 | TN | [4] |
|  | Lys97Val | Decreased | 9ILB (A) | TN | AF-P01584-F1 | TN | [4] |
| Apolipoprotein D | Trp99His  Ile118Ser  Leu120Ser | Increased | 2HZR (A) | TP | AF-P05090-F1 | TP | [5] |
| Leptin | Trp100Glu | Increased | 1AX8 (A) | TP | AF-P41159-F1 | TP | [6] |
|  | His97Ser  Trp100Gln  Ala101Thr  Gly112Glu  Met136Ile  Trp138Gln  Gly145Glu | Increased | 1AX8 (A) | TP | AF-P41159-F1 | TP | [6] |
|  | Trp100Gln  Trp138Gln | Increased | 1AX8 (A) | TP | AF-P41159-F1 | TP | [6] |
| Abbreviations: TP, True positives; TN, true negatives; PDB code, Protein Data Bank accession number; AF code, Alpha Fold Protein Structure Database accession number. | | | | | | | |

**3. A3D-DB use considerations**

A3D is a structure-based algorithm that uses three-dimensional protein coordinates to project each amino acid's intrinsic aggregation propensity value into the structure. Then, the aggregation score is corrected as a function of its specific solvent exposure and the aggregation propensity of neighboring residues within a 10 Å sphere of radius. Given this dependence on the spatial position of each atom of the protein, both the atomic resolution and the biological relevance of the input structures impact A3D prediction's accuracy. Therefore, in order to correctly interpret A3D database aggregation tendencies, users might consider two critical characteristics of the AF database: (i) structure confidence and disorder content and (ii) quaternary structure context.

**3.1 Interpreting structure confidence and disorder content**

AF predicted structures cover 98.5% of the human proteome and involve complete protein chains in their monomeric state. Each amino acid in an AF model has a confidence score (pLDDT) that ranges from 0 to 100, indicating which regions could be considered equivalent to an experimentally determined structure. It is estimated that around 30% of the amino acids of the human proteome are located outside globular (well-folded) domains and constitute intrinsically disordered or low-complexity regions. Therefore, it is not surprising that AF-predicted structures frequently contain regions with very low (pLDDT < 50) scores [(Ruff and Pappu, 2021)](https://paperpile.com/c/2SWuDF/dEc5E). These permanently or transiently disordered segments are often not defined in experimental structures, and therefore were excluded from the A3D analysis. Now they are incorporated in the A3D Database. The A3D predictions are blind to the dynamic nature of these regions since AF models correspond to static single frames of the conformational ensemble. Therefore, the presence of disordered regions with low AF confidence might artificially shelter or increase the presence of S-APRs in adjacent high confidence protein domains.

To deal with this issue, the A3D dataset includes two additional precalculated structural aggregation profiles, which correspond to predicted structures with the pLDDT thresholds: pLDDT > 70 and pLDDT > 50 confidence, which was defined after manually curating the 100 AF models depicted in Table S1.

In the following points, we outline different situations users may face:

- Proteins with high confidence values: the overall AF-predicted structure possesses high pLDDTs. All three computed structures converge into a unique aggregation prediction. Well-defined S-APRs can be delineated from A3D output structures (representative examples: Q68D91, S4R460, see panel a) in **Figure S8**).
- Proteins with limited low confidence regions: proteins that display local surface modifications with confidence thresholds of >70% or >50% pLDDT. An increase or decrease of localized S-APRs can be observed (representative examples: P09382, Q9UKL6, Q5EE01, see panel d) in **Figure S8**).
- Multidomain proteins: proteins with two or more globular domains that correspond to well-defined and confident regions, while tethering elements that connect globular domains possess low pLDDT values. The analysis with restricted pLDDT thresholds often results in disconnected domains and, as a consequence, some previously sheltered APRs might become exposed. As a result, S-APRs might diverge in the tree models, and their boundaries are less evident (representative examples: Q96MN5, Q96DA0, see panel b) in Figure 5). Note that for each database entry, the AlphaFold database provides Predicted Aligned Error (PAE) analysis. The PAE is useful for assessing whether relative domain positions are predicted correctly or not. If not, A3D analysis may be affected by incorrectly exposed domains.
- Proteins with extended low-confidence regions: proteins with large low confidence regions localized in flexible C-, N-terminus, or long loops, usually displayed around a well-defined globular core. With the 70% and 50% pLDDTs cutoffs, disordered regions are often excluded from the model and, therefore, not considered in A3D prediction. As a result, the A3D predictions correspond to well-folded globular domains. Note that we might observe the presence of free amino acids in some cases, unattached to the protein main chain, essentially because they are in the exclusion limit between two pLDDT thresholds (representative examples: Q9NRE2, Q8NFW1, see panel e) in **Figure S8**).
- Proteins with overall low confidence: usually, short polypeptides that are mostly disordered, exposing most of its surface to solvent. In some cases, we lose all the structure in predictions corresponding to >70% or >50% pLDDT threshold. (representative examples: Q8N9P0, Q8TEV8, Q9GZY1, see panels c) and f) in **Figure S8**).


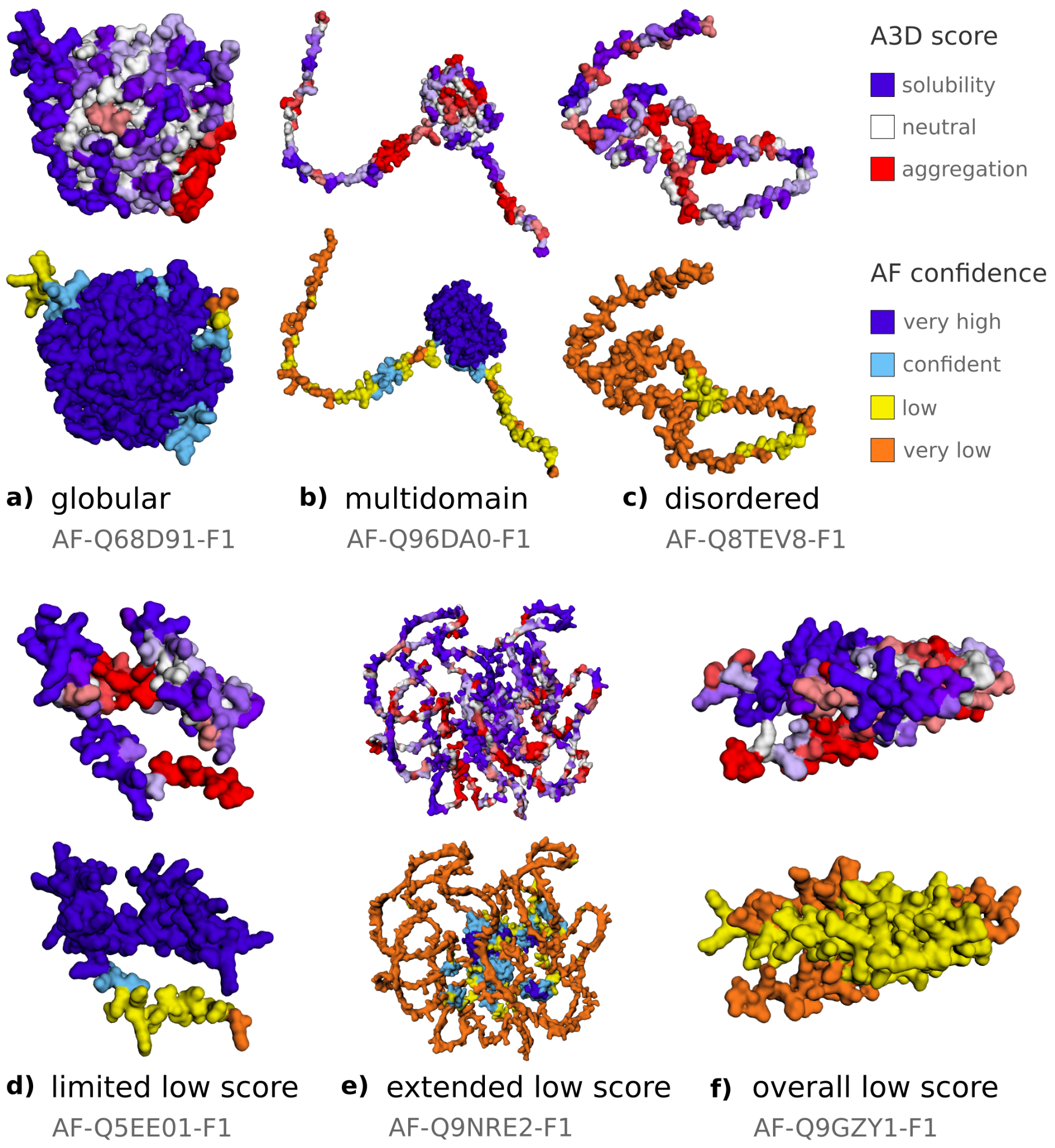


**Figure S8.** An overview of the different types of cases that may be encountered in the A3D database. The presented cases are colored using A3D score (top panels) and AF confidence score (bottom panels) and discussed in the 4.1 section.

**3.2 Interpreting quaternary structure context**

In the AF database, structure predictions are restricted to single chains. This precludes the analysis of the quaternary structure context in the A3D Database. The overlap between the physicochemical properties governing protein-protein native interactions and non-native contacts triggering aberrant self-assembly implies that protein interfaces are enriched in S-APRs. In proteins displaying quaternary structure, this results in an over-prediction of S-APRs in protein monomers, relative to native the same subunits in the oligomeric state. Thus, when the formation of quaternary structure is suspected the user is advised to run the multimer version of AF locally and, once validated, to employ the resulting PDB of the generated model as an input in A3D.

**3.3 Guideline for A3D database analysis**

In this work, we suggest a general guideline to manage the A3D output. In addition, we strongly encourage users to compile as much information about their case study protein to exploit A3D precalculated predictions. Some helpful questions to shape the output would be:

1. Does the protein possess disordered regions?
2. Do these disordered segments correlate with low pLDDT?
3. Does the protein bind another protein? Does it form a homo- or hetero-oligomer?
4. Does the protein contain a signal peptide?
5. Is the protein the mature form or on the contrary, is a proprotein?

In addition to the aforementioned pieces of advice, we also recommend reviewing dedicated bibliography on how to manage the A3D algorithm and how to evaluate its aggregation predictions [(Pujols *et al.*, 2018; Kuriata *et al.*, 2019; Zambrano *et al.*, 2015; Santos *et al.*, 2020)](https://paperpile.com/c/2SWuDF/FaW1n+NbUJq+tGjOh+P4p7L).

**SUPPLEMENTARY REFERENCES**

[Adachi,K. *et al.* (1993) Effects of beta 6 aromatic amino acids on polymerization and solubility of recombinant hemoglobins made in yeast. *J. Biol. Chem.*, **268**, 21650–21656.](http://paperpile.com/b/2SWuDF/jybo)

[Akdel,M. *et al.* (2021) A structural biology community assessment of AlphaFold 2 applications. *bioRxiv*.](http://paperpile.com/b/2SWuDF/2nMSW)

[Camilloni,C. *et al.* (2016) Rational design of mutations that change the aggregation rate of a protein while maintaining its native structure and stability. *Sci. Rep.*, **6**, 25559.](http://paperpile.com/b/2SWuDF/WguV)

[Deng,H.-X. *et al.* (1993) Amyotrophic Lateral Ssclerosis and Structural Defects in Cu,Zn Superoxide Dismutase. *Science*, **261**, 1047–1051.](http://paperpile.com/b/2SWuDF/qVYwa)

[Elam,J.S. *et al.* (2003) Amyloid-like filaments and water-filled nanotubes formed by SOD1 mutant proteins linked to familial ALS. *Nat. Struct. Biol.*, **10**, 461–467.](http://paperpile.com/b/2SWuDF/xo0jY)

[Gejyo,F. *et al.* (1985) A new form of amyloid protein associated with chronic hemodialysis was identified as beta 2-microglobulin. *Biochem. Biophys. Res. Commun.*, **129**, 701–706.](http://paperpile.com/b/2SWuDF/AGIF)

[Ito,T. and Wagner,G. (2004) Using codon optimization, chaperone co-expression, and rational mutagenesis for production and NMR assignments of human eIF2α. *J. Biomol. NMR*, **28**, 357–367.](http://paperpile.com/b/2SWuDF/8SAh)

[Jumper,J. *et al.* (2021) Highly accurate protein structure prediction with AlphaFold. *Nature*, **596**, 583–589.](http://paperpile.com/b/2SWuDF/qN6VB)

[Kuriata,A. *et al.* (2019) Aggrescan3D (A3D) 2.0: prediction and engineering of protein solubility. *Nucleic Acids Res.*, **47**, W300–W307.](http://paperpile.com/b/2SWuDF/NbUJq)

[Murphy,R. and Tsai,A. (2007) Misbehaving Proteins: Protein (Mis)Folding, Aggregation, and Stability Springer Science & Business Media.](http://paperpile.com/b/2SWuDF/2zbF)

[Nasreen,A. *et al.* (2006) Solubility engineering and crystallization of human apolipoprotein D. *Protein Sci.*, **15**, 190–199.](http://paperpile.com/b/2SWuDF/r7wD)

[Pujols,J. *et al.* (2018) AGGRESCAN3D: Toward the Prediction of the Aggregation Propensities of Protein Structures. *Methods Mol. Biol.*, **1762**, 427–443.](http://paperpile.com/b/2SWuDF/FaW1n)

[Ruff,K.M. and Pappu,R.V. (2021) AlphaFold and Implications for Intrinsically Disordered Proteins. *J. Mol. Biol.*, **433**, 167208.](http://paperpile.com/b/2SWuDF/dEc5E)

[Santos,J. *et al.* (2020) Computational prediction of protein aggregation: Advances in proteomics, conformation-specific algorithms and biotechnological applications. *Comput. Struct. Biotechnol. J.*, **18**, 1403–1413.](http://paperpile.com/b/2SWuDF/P4p7L)

[Schymkowitz,J. *et al.* (2005) The FoldX web server: an online force field. *Nucleic Acids Res.*, **33**, W382–8.](http://paperpile.com/b/2SWuDF/1UBEi)

[Sim,J. and Sim,T.-S. (1999) Amino acid substitutions affecting protein solubility: high level expression of Streptomyces clavuligerus isopenicillin N synthase in Escherichia coli. *J. Mol. Catal. B Enzym.*, **6**, 133–143.](http://paperpile.com/b/2SWuDF/K51c)

[Sun,Z.Y. *et al.* (1999) Functional glycan-free adhesion domain of human cell surface receptor CD58: design, production and NMR studies. *EMBO J.*, **18**, 2941–2949.](http://paperpile.com/b/2SWuDF/kCcT)

[Tsirigos,K.D. *et al.* (2015) The TOPCONS web server for combined membrane protein topology and signal peptide prediction. *Nucleic Acids Res.*, **43**, W401–W407.](http://paperpile.com/b/2SWuDF/xH7H)

[Zambrano,R. *et al.* (2015) AGGRESCAN3D (A3D): server for prediction of aggregation properties of protein structures. *Nucleic Acids Res.*, **43**, W306–13.](http://paperpile.com/b/2SWuDF/tGjOh)
